# Disease Risk and Conservation Implications of Orangutan Translocations

**DOI:** 10.3389/fvets.2021.749547

**Published:** 2021-11-12

**Authors:** Julie Sherman, Steve Unwin, Dominic A. Travis, Felicity Oram, Serge A. Wich, Ricko L. Jaya, Maria Voigt, Truly Santika, Emily Massingham, Dave J. I. Seaman, Erik Meijaard, Marc Ancrenaz

**Affiliations:** ^1^Wildlife Impact, Portland, OR, United States; ^2^School of Biosciences, University of Birmingham, Birmingham, United Kingdom; ^3^One Health Division, Department of Veterinary Population Medicine, College of Veterinary Medicine, University of Minnesota, St. Paul, MN, United States; ^4^Pongo Alliance-Kinabatangan, Kota Kinabalu, Malaysia; ^5^School of Biological and Environmental Sciences, Liverpool John Moores University, Liverpool, United Kingdom; ^6^Orangutan Information Centre, Medan, Indonesia; ^7^Durrell Institute of Conservation and Ecology, School of Anthropology and Conservation, University of Kent, Canterbury, United Kingdom; ^8^Natural Resources Institute, University of Greenwich, Chatham, United Kingdom; ^9^School of Biological Sciences, University of Queensland, St Lucia, QLD, Australia; ^10^Borneo Futures, Bandar Seri Begawan, Darussalam, Brunei; ^11^HUTAN, Sandakan, Malaysia

**Keywords:** translocation, reintroduction, reinforcement, COVID-19, orangutan, conservation, disease

## Abstract

Critically Endangered orangutans are translocated in several situations: reintroduced into historic range where no wild populations exist, released to reinforce existing wild populations, and wild-to-wild translocated to remove individuals from potentially risky situations. Translocated orangutans exposed to human diseases, including Coronavirus Disease 2019 (COVID-19), pose risks to wild and previously released conspecifics. Wildlife disease risk experts recommended halting great ape translocations during the COVID-19 pandemic to minimize risk of disease transmission to wild populations. We collected data on orangutan releases and associated disease risk management in Indonesia during the COVID-19 pandemic, and developed a problem description for orangutan disease and conservation risks. We identified that at least 15 rehabilitated ex-captive and 27 wild captured orangutans were released during the study period. Identified disease risks included several wild-to-wild translocated orangutans in direct contact or proximity to humans without protective equipment, and formerly captive rehabilitated orangutans that have had long periods of contact and potential exposure to human diseases. While translocation practitioners typically employ mitigation measures to decrease disease transmission likelihood, these measures cannot eliminate all risk, and are not consistently applied. COVID-19 and other diseases of human origin can be transmitted to orangutans, which could have catastrophic impacts on wild orangutans, other susceptible fauna, and humans should disease transmission occur. We recommend stakeholders conduct a Disease Risk Analysis for orangutan translocation, and improve pathogen surveillance and mitigation measures to decrease the likelihood of potential outbreaks. We also suggest refocusing conservation efforts on alternatives to wild-to-wild translocation including mitigating human-orangutan interactions, enforcing laws and protecting orangutan habitats to conserve orangutans *in situ*.

## Introduction

Translocation is the human movement of wildlife between habitats or from captive facilities into natural habitats. Translocation is widely practiced to recover wild populations, release animals confiscated from the illegal wildlife trade, and address negative human-wildlife interactions (1, 2). Great apes—chimpanzees, gorillas, bonobos, and orangutans—are among the taxa translocated in these circumstances (3). Translocation is an especially common practice in Indonesian orangutan management (4). Orangutans in Indonesia are translocated in two situations: (1) wild orangutans are captured due to actual or potential conflict with humans, perceived isolation in forest fragments, or rescue from forest fires, and moved to a new location immediately or within a short period (wild-to-wild translocation); and (2) individuals rescued from illegal trade or captivity are rehabilitated and released to supplement wild populations (reinforcement) or re-establish populations within historic range (reintroduction) (4, 5).

Bornean orangutans (*Pongo pygmeaus*), Sumatran orangutans (*Pongo abelii*) and Tapanuli orangutans (*Pongo tapanuliensis*) are listed as Critically Endangered by the International Union for Conservation and Nature (IUCN) Red List of Threatened Species (6–8). Orangutan conservation efforts are confounded by a range of anthropogenic factors including poverty among humans sharing orangutan habitats, high demand for use of these habitats for agriculture and natural resource extraction, movement of humans into orangutan habitats, and human-orangutan interactions and conflicts (9). Due to these many deeply entrenched issues, orangutan conservation has been described as a “wicked complex” problem that cannot be easily resolved (10, 11). Over the past several decades, translocation has been embraced as a means to protect individual orangutans by moving them away from areas of human use or habitation, and as a conservation strategy to re-establish or reinforce wild populations (4).

IUCN has published best practice guidelines for wildlife translocations, including for great apes (1, 3). The precautionary principle for any great ape release requires that, above all, it must not endanger resident wild populations via communicable disease, hybridization, excessive social disruption or exacerbated competition for resources (3). IUCN guidelines further stipulate that individual welfare benefits alone are not considered a valid rationale for a conservation release and that conservation of the taxon and wild conspecifics takes precedence over the welfare of captive individuals (1, 3). IUCN is not a regulatory body, hence guidelines are only enforceable if mandated by government or local decision makers. The 2007–2017 Indonesian Orangutan Action Plan and the draft 2019–2029 plan refer to IUCN guidelines and incorporate some of their recommendations, including avoiding release of orangutans with infectious diseases into wild populations (12, 13).

Although translocation is an important conservation tool for many species (1, 14), there are increasing concerns about its effectiveness for orangutans. This is due to the risks that translocation poses to wild populations and its use of funds and political will, which might otherwise be available for habitat conservation and anti-poaching efforts. Specific risks of translocation for orangutans include: disease transmission, competition and social stress for released and wild orangutans; suspected high incidences of mortality following release; and negative impacts on genetic and socioecological functioning of the source populations if individuals are removed through wild-to-wild translocation (4, 5, 15–18). While translocations are never entirely without risk, infectious disease transmission from released animals to wild populations can pose particularly consequential risks to wild individuals and to population health (19). These risks exist even if few orangutans are translocated, but are compounded as translocation numbers rise.

The potential for released individuals to transmit disease to wild populations is particularly pertinent as the world struggles to contain the COVID-19 pandemic. Being our close living relatives, non-human primates are particularly sensitive to human communicable diseases (20, 21). Wild and captive apes in proximity to researchers, caregivers, tourists, and community members can easily contract spillover diseases of human origin (21–24). Consequently, releases of apes into natural habitats can expose resident wild populations to significant health risks (25–27). The COVID-19 pandemic is an example of one such disease of concern. The disease, caused by a coronavirus, SARS-CoV-2, has infected captive gorillas and several other wildlife taxa, and likely can infect orangutans and other primates (28–33). Gorillas in at least two zoos have contracted COVID-19 from caregivers, despite the caregivers wearing masks, observing other COVID-19 safety protocols, and being asymptomatic (28, 34). This risk is increased by recent COVID-19 variants including the more transmissible Delta variant, which has already infected several zoo gorillas, likely via a fully vaccinated but infected and asymptomatic keeper who was observing all safety protocols (35).

For this study, we sought to answer the questions, “Can orangutan translocation pose infectious disease transmission and species conservation risks?,” and “What are the implications of these risks in the COVID-19 era?” To address these questions, we collected publicly reported and unpublished data on orangutan translocations in Indonesia between March 15, 2020 and March 14, 2021, the period covering the first two waves of COVID-19 human infection in the country. We reviewed these records on wild-to-wild translocations and releases of rehabilitated orangutans to identify trends in translocation practice and disease risk management during the COVID-19 pandemic. We considered peer-reviewed published literature and the authors' unpublished data to assess best available evidence on orangutan conservation priorities and challenges, and the health and conservation impacts of orangutan translocation. We used these translocation, health, and conservation data and the World Organization for Animal Health (OIE) and IUCN wildlife disease risk procedures (36) to develop a problem description and a list of potential disease risk pathways in orangutan translocations. This problem description identifies risks specific to the three different types of translocations practiced with orangutans: reinforcements, reintroductions and wild-to-wild translocations. The problem description is the precursor to a full Disease Risk Analysis (DRA) for orangutan translocations, which is planned as part of an orangutan translocation practitioner and conservation stakeholder workshop later in 2021. Finally, we present a qualitative logic matrix, employing COVID-19 as an example of pathogen transmission risk, which can be used or adapted to weigh relative health and conservation risks of orangutan translocation. These considerations are applicable across great ape taxa and to many other non-human primate species.

## Materials and Methods

### Orangutan Translocation Data Collection and Analysis

We collected data on orangutan translocation events between March 15, 2020 and March 14, 2021. To obtain these data we reviewed online newspapers, government reporting, and reporting by non-governmental organizations (NGOs) focused on orangutan rescue, rehabilitation and release. We searched the department of Natural Resources and Ecosystem Conservation [*Konservasi Sumber Daya Alam dan Ekosistem* (KSDAE)] newsblog, and Indonesian newspaper websites ANTARA News, Prokal, TribunNews, Kompas, and The Jakarta Post, using the search term “orangutan” to capture any relevant news published during the study period. We also performed a Google Advanced Search for news results in both English and Bahasa Indonesia for the search term “orangutan” published during this period. The term “orangutan” is typically spelled the same in both languages, although searches for “orangutan” also returned results for the alternate spelling “orang-utan.” We compiled reported information from the ten organizations that held and released orangutans within Indonesia as of 2020, namely Bornean Orangutan Survival Foundation, Centre for Orangutan Protection/Borneo Orangutan Rescue Alliance, International Animal Rescue-Ketapang, Orangutan Foundation International, Orangutan Foundation–UK, Orangutan Information Centre, Sintang Orangutan Centre, Yayasan Jejak Pulang, and government facility BKSDA Tenggarong. Provincial departments of KSDAE, the *Balai Konservasi Sumber Daya Ekosistem* (BKSDA) also rescue and release orangutans and may temporarily hold them but do not operate long-term captive care facilities. Data on BKSDA releases were collated from the KSDAE online newsblog, and from online newspaper sources. Data on NGO releases were collected from these organizations' newsblogs, press releases, social media posts, annual reports, monitoring reports. Articles in Bahasa Indonesia were translated using Google translate, and we referred questions on meaning or nuance to Indonesian speaking authors of this paper.

For each record referencing orangutan translocations, we extracted any available data on: (1) rescue and release dates; (2) number of animals; (3) individual animal's age, sex, name, physical condition; (4) any description of health checks and post-release monitoring; (5) the origin and release locations; and (6) the entities conducting the translocation. Wherever possible we analyzed multiple sources to confirm data and address gaps within individual records. We excluded any possible duplicate records by reviewing for repeat mentions of combinations of available information on release date; animal name, age, and sex; and the translocation location and circumstances. We excluded all records where it was unclear if the animal had been previously counted. Releases were classified as: (1) reintroduction/reinforcements if they involved ex-captive orangutans that spent 6 months or more in rehabilitation facilities, or (2) wild-to-wild translocation if they involved wild orangutans captured and released within six months or less. Re-releases of previously released and recaptured orangutans were classified as the type of release originally conducted.

Release rates are the annual sums of individual animals released by release classification. For comparison of release rates before the COVID-19 pandemic, we used our published data on orangutan rescues and releases from 2007–2017 (4), and authors' dataset on 2018–2019 rescues and releases (Sherman, Ancrenaz and Meijaard unpublished data, Jaya unpublished data). The collection methods for these data are the same as described here for 2020 data.

### Problem Description and Qualitative Logical Risk Assessment Matrix

The authors comprise a group of wildlife health, conservation and orangutan experts including orangutan translocation practitioners, orangutan conservation practitioners, and specialists in orangutan population, socioecology, health, and welfare. These practitioners considered the research questions of whether orangutan translocations can pose disease transmission and conservation risks, and specifically COVID-19 transmission risks. We followed the problem description questions posed by the OIE and IUCN Manual on Procedures for Wildlife Disease Risk Analysis (2014) in considering these queries. A problem description identifies the questions and issues being considered, assesses the cultural, political and social contexts that affect these issues, and identifies potential pathways by which risk is introduced (37). Here, we reviewed data to identify four elements of potential risk: (1) is the problem (pathogen transmission and conservation risk) potentially occurring?; (2) in which ways could the problem occur?; (3) what is the scope and context of the problem?; and (4) how should the problem description inform action? Some authors of this paper will also participate in the upcoming orangutan Disease Risk Analysis workshops, while others are not part of the workshop group, and serve here to provide external, independent views on the problem description. Having separate and independent problem description and hazard identification/DRA processes has been recommended to prevent political and social biases (38).

To determine whether transmission is potentially occurring (element 1 of potential risks, as described above), we reviewed the available data on orangutan translocations during the COVID-19 era (Orangutan Translocation Data Collection and Analysis). To examine which ways the problem could occur (element 2), we used published literature and author's expert knowledge to identify the “risk pathways” –logical routes by which pathogens could be introduced to orangutans from humans, or vice versa, or could spread amongst orangutans in captivity or in the wild (Table 1). It was necessary to answer OIE and IUCN (36) problem description questions separately for each translocation type (reinforcement, reintroduction, and wild-to-wild translocation), as each translocation type has a distinct set of risks and uncertainties. Element 3 required consideration of the conservation, policy and cultural contexts underpinning orangutan translocations, using published literature and author's unpublished data. Elements 1–3 are summarized in Table 2, a matrix of answers to the OIE and IUCN (36) problem description questions.

**Table 1:** Pathogen risk pathways in orangutan rescue, rehabilitation and translocation.

**Translocation activity**	**Potential pathogen risk pathways**	**Activity relevance bytranslocation type**
Orangutan interactions or conflict with humans; capture and translocation of wild orangutans encountered in habitats occupied or regularly used by humans	• Human proximity or direct contact from tourism, research, or local communities in shared habitats • Injury or contact with human equipment or other possible fomites • Wildlife vectors in human-occupied habitats (rodents, insects, etc.) • Human, pet or livestock waste or other infected matter	Wild-to-wild
Time in human captivity (other than at rescue center)	• Prolonged human proximity and direct contact with humans • Prolonged proximity with other captive apes that are potentially a source of pathogens • Exposure to or direct contact with livestock or pets • Injury or contact with human equipment or other possible fomites • Stress leading to increased susceptibility or recurrence of latent disease • Wildlife vectors in human residences or businesses (rodents, insects, etc.) • Human, pet or livestock waste or other infected matter	Reinforcement; Reintroduction
Intervention to move orangutans in conflict situations; darting and capture for wild-to-wild translocation	• Prolonged human proximity and direct contact with humans • Injury or contact with human equipment or other possible fomites • Stress leading to increased susceptibility or recurrence of latent disease	Wild-to-wild
Live capture by hunters or in snare	• Human proximity and direct contact with humans • Injury or contact with human equipment or other possible fomites	Wild-to-wild; Reinforcement; Reintroduction
Physical examination and emergency care	• Human proximity and direct contact with humans • Exposure to or direct contact with humans and other wildlife • Injury or contact with human equipment or other possible fomites	Wild-to-wild; Reinforcement; Reintroduction
Transport to care facility	• Human proximity and direct contact with humans • Injury or contact with human equipment or other possible fomites	Wild-to-wild; Reinforcement; Reintroduction
Captive care and rehabilitation in rescue center	• Prolonged human proximity and direct contact with humans, including staff, volunteers, researchers and tourists • Exposure to or direct contact with conspecifics, other wildlife, and pets • Injury or contact with human equipment or other possible fomites • Stress leading to increased susceptibility or recurrence of latent disease • Wildlife vectors in captive environments (rodents, insects, etc.) • Human, pet, livestock, or other captive center wildlife waste or infected matter	Reinforcement; Reintroduction
Transport to release site	• Human proximity and direct contact with humans • Injury or contact with human equipment or other possible fomites	Wild-to-wild; Reinforcement; Reintroduction
Release	• Human proximity and direct contact with humans • Direct contact with conspecifics and other wildlife • Exposure to novel pathogens in new habitats • Introduction of novel pathogens to resident conspecific populations	Wild-to-wild; Reinforcement; Reintroduction
Post-release	• Direct contact with conspecifics and other wildlife • Exposure to novel pathogens in new habitats • Introduction of novel pathogens to resident conspecific populations • Proximity or interactions with humans and human equipment during PRM activities and provisioning	Wild-to-wild; Reinforcement; Reintroduction

*Rescue, rehabilitation and release activities associated with potential pathogen risk pathways*.

**Table 2:** Orangutan disease risk problem description.

**Question**	**Summary description**	**References**
What is the nature of the problem?	• Pathogen risk mitigation strategies do not appear to be consistently applied; lack of crowd control at rescue and release events, lack of PPE use, and limited pathogen surveillance pose notable risks of pathogen transfer to rehabilitant and wild populations • Orangutans are Critically Endangered. Deforestation, habitat fragmentation and killing are most pressing needs • Translocation is often viewed as the preferred solution despite higher cost and risks • Diverse stakeholder needs • Wicked complexity of orangutan conservation issues	(5), (9, 10), (39), (40, 41), (42)
What are the management goals and decisions needed? How will the risk analysis help?	Goals: • Design and implement regular surveillance of pathogens in wild, captive, and translocated orangutans • Quantify the risk of pathogen transfers in orangutan translocation • Improve viability of wild orangutan populations • Improve protection of habitats, biodiversity and human safety	
	Risk analysis benefits: • Determine circumstances wherein translocation is likely to provide a net benefit to orangutan conservation that outweighs conservation and biosecurity risks • Develop and implement appropriate measures to limit disease risk • Beta test the pathogen transfer management system for diseases of concern including COVID-19 • Promote regime of accurate sampling to avoid false negatives due to poor sampling techniques and to create stored samples for further analysis	
What is the ecological level of concern (population, community, ecosystem)?	All orangutan species are Critically Endangered, and all populations are at risk from killing, deforestation, and habitat degradation and fragmentation. Orangutan metapopulations are at risk from these same factors and may be harmed by removal of animals by wild-to-wild translocation. In areas of orangutan distribution where habitats have been degraded, fragmented or deforested, ecosystem functions are likely also compromised	(5), (40, 41), (43), (42)
Are there any policy or regulation considerations?	Government policy calls for: • Preventing loss of orangutans across all habitats • Translocation of all releasable rehabilitated orangutans • Use of wild-to-wild translocation only as a last resort	(12), (13)
What precedents are set by similar DRAs and previous decisions?	• Decision by practitioners at OVAG: no wild-to-wild translocations during COVID-19 pandemic • IUCN SGA guidance: no wild-to-wild translocation during COVID-19 pandemic; improve pathogen transmission risk management for any activities involving great apes	(32); Unwin unpublished data
What is the cultural and political history and current context of the problem as represented through the eyes and values of different stakeholders?	• Wildlife health experts are concerned about: (1) effectiveness of pathogen risk mitigation strategies as currently implemented, and how these strategies are evaluated; (2) lack of resources to allow sample storage and analysis; and (3) reluctance on part of practitioners to collect samples at time of translocation as a baseline. Specific concerns for pathogen transfer include lack of official crowd control and prevalence of people with no PPE in contact or <6 foot distance to orangutans for >15 min potential exposure time, lack of sampling for surveillance and probabilities of zoonotic transmission based on data from other great apes • Orangutan scientists and conservationists are concerned about: (1) lack of demonstrated evidence of population and species-level benefits from wild-to-wild translocations and reinforcements of likely viable wild populations; (2) weak law enforcement–only a small fraction of orangutan habitat is covered by patrols, rules are not enforced consistently and prosecution and sentencing for crimes affecting orangutans are exceptionally rare; (3) there are conflicting policies on promoting land development and protecting rare species habitat, and (4) orangutan conservation funding and activities to date have proven insufficient for species recovery • Local community members living in orangutan range who suffer from poverty, insufficient well-being, and lack of livelihood opportunities feel conservation regulations and practices indicate orangutans are valued more than people	(4, 5), (9, 10, 39), (44), (42), Santika et al., in prep.
	• Translocation practitioners and government are concerned that: (1) orangutans in potential conflict with humans may be harmed or killed; (2) orangutans encountered in forest fragments or in agricultural plantations may suffer from lack of food or poor nutrition; (3) lack of space in rehabilitation facilities; and (4) orangutans living in plantations, forest fragments, or in rehabilitation facilities may have poorer welfare than those living in large protected forests	
What resources (e.g., personnel, time, money) are needed and available?	• Investments in orangutan translocation are substantial, but significantly increased resources are needed for long term monitoring, development and implementation of solutions for human-orangutan coexistence, as well as for disease surveillance • More buy in is needed from translocation practitioners for significant increases in surveillance and disease testing • Equipment, funding and other resources for pathogen surveillance are needed locally. While laboratories and equipment are available internationally, the organizations implementing translocations locally cannot always access them. Capacity building, financial and equipment resources, personnel and staff time are needed for sampling and sample storage	(44), (45), Santika et al., in prep.
What level of risk is acceptable?	Views on acceptable risk differ among stakeholders. Wildlife health experts, orangutan scientists and conservationists urge a precautionary approach that would involve increased surveillance, a DRA, consistently applied risk mitigation measures, and cessation of most wild-to-wild translocations due to high risk and uncertain benefits. Most translocation practitioners are comfortable with the risks posed by current translocation practices	Authors' unpublished data, (4), (46)
What documents or data exist to describe the state of knowledge of the problem?	See references cited for this paper. Data are available on some aspects of orangutan socioecology and habitat use, population status, zoonotic disease risks, and the outcomes and impacts of translocation and other conservation interventions	

*Questions from OIE and IUCN Manual for Procedures for Wildlife Disease Risk Analysis (2014). Summary descriptions for each question are based on data collected for this paper, and published and unpublished sources listed*.

To exemplify how the problem description could inform decision making and action (element 4), we used SARS-CoV-2—an example of a human to non-human primate transmissible pathogen—to develop the qualitative risk matrix tool (**Figure 5**). We considered published data on COVID-19 disease presence in humans and great apes, and in other wildlife and domestic animals. We used qualitative categories of risk because data were not available for captive and wild orangutan exposure and transmission of the SARS-CoV-2 pathogen in Indonesia, and to make the matrix more useful to the diverse array of orangutan translocation stakeholders and decision makers (38). We made a logical stepwise matrix of risk categories based on the likelihood of active infection; exposure of susceptible individuals; and consequences of susceptible recipients becoming infected, getting sick or dying, and spreading the disease (28, 32). The risk matrix is based on Risk = Likelihood × Consequences. Likelihood and consequence category definitions are provided in the cells bounding the risk matrix. Category definitions are based on human health and safety risk likelihood and consequence definitions (47, 48). We also factored in mitigations including personal protective equipment (PPE), disease testing, and vaccination, as well as uncertainties about disease transmission and its consequences (28, 33). We weighed associated conservation risks using the IUCN precautionary principles, which state that great ape releases should not endanger wild conspecifics, other taxa within the release habitat, or habitat ecosystem functions (1, 3).

## Results

### Orangutan Translocations During COVID-19 Pandemic

Our results suggest that the problem—pathogen transmission between humans and orangutans—is potentially occurring. Orangutans are being handled by or are in physical proximity with humans for extended periods. Translocated orangutans are highly likely to be in contact with wild orangutans after release. Other than the two sites in Sumatra, where wild-to-wild translocated and rehabilitated orangutans are reintroduced within historical range but outside current distribution (49), orangutans are purposefully released into wild populations, or into areas where they have the potential to disperse into existing wild populations (4, 50, 51).

**Figure 1:**
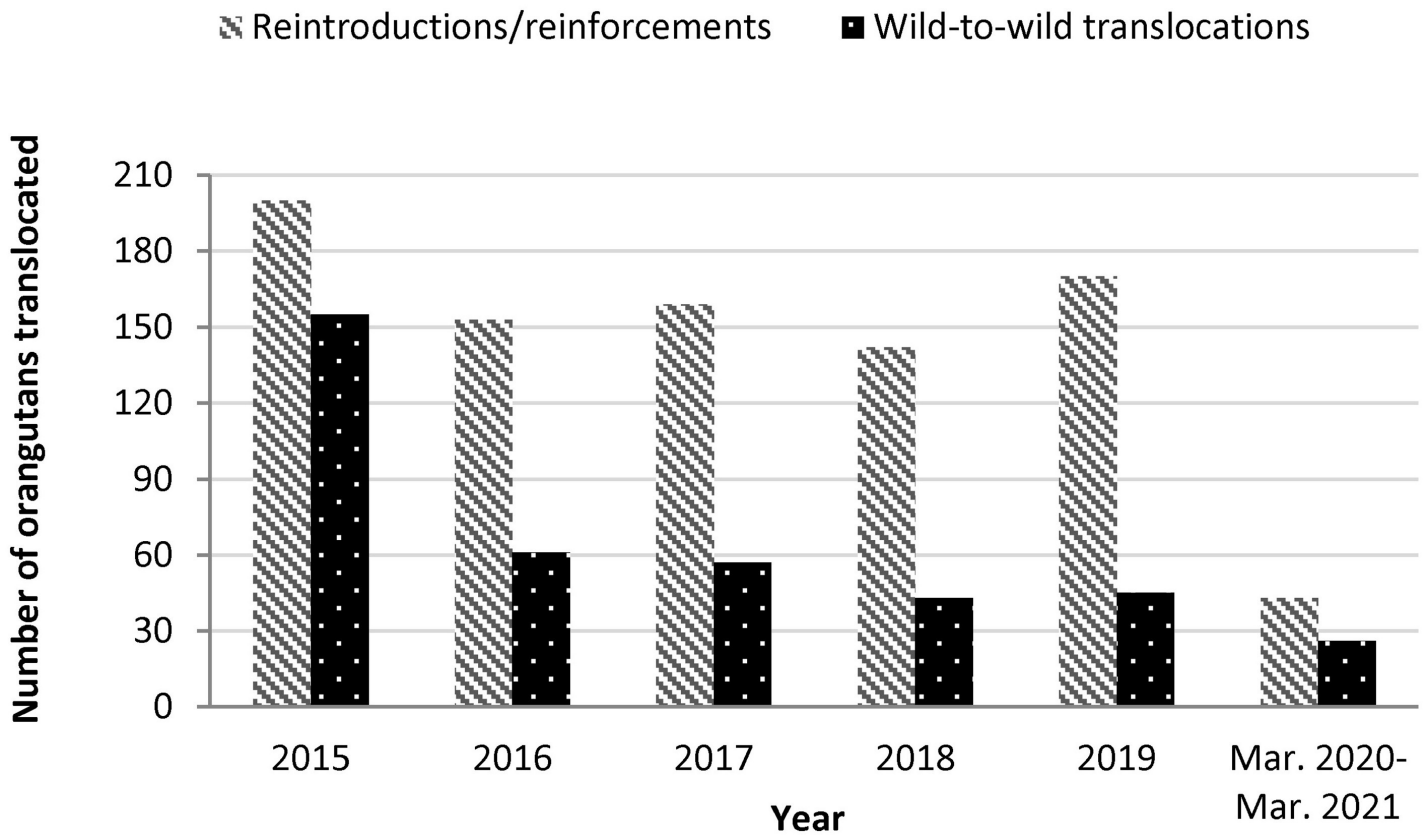
Orangutan translocations in Indonesia since 2015. Annual rates are estimated from Sherman, Ancrenaz and Meijaard (4), available rescue center annual reports and news blogs, KSDAE news blog and unpublished data. Release rates shown may be lower than actual releases, as some rescue centers did not have publicly available data for all years.

Orangutan translocations decreased in frequency but continued throughout the COVID-19 pandemic. We identified releases of at least 15 rehabilitated ex-captive orangutans (reintroductions or reinforcements), and 27 wild-to-wild translocations in Indonesia from March 15, 2020 to March 14, 2021 (Table 3). At least two wild-to-wild translocations occurred during the March–May 2020 Indonesian government moratorium on captive wildlife releases. Three wild orangutans, one *P. pygmaeus*, one *P. abelii* and one *P. tapanuliensis*, were translocated multiple times during the study period. Total orangutan releases declined 74% and wild-to-wild translocations declined 64% during the study period compared to the average annual rate over the 5 years before COVID (2015–2019) (Figure 1). Notably the number of wild-to-wild translocations was highest in 2015, likely induced by the greater incidence of fires during the El Nino phase that year. The annual wild-to-wild translocation rate dropped from 155 in 2015 to 61 in 2016, and varied between 57 and 26 between 2017 and 2019.

**Table 3:** Orangutan translocations during the COVID-19 pandemic in Indonesia.

**Event #**	**Species**	**Translocation type**	**Animal source**	**# OU released**	**Date**	**Reference**
1	*P. pygmaeus*	Wild-to-wild	Wild captured	3	23 Mar. 2020	(52)
2	*P. abelii*	Wild-to-wild	Recapture of wild OU	1	20 Apr. 2020	(53)
3	*P. abelii*	Wild-to-wild	Recapture of wild OU	1	7 Jul. 2020	(54)
4	*P. pygmaeus*	Wild-to-wild	Wild captured	1	24 Aug. 2020	(55)
5	*P. abelii*	Wild-to-wild	Wild captured	1	15 Sept. 2020	(56)
6	*P. abelii*	Wild-to-wild	Wild captured	1	15 Oct. 2020	(57)
7	*P. tapanuliensis*	Wild-to-wild	Recapture of wild OU	1	23 Nov. 2020	(58)
8	*P. pygmaeus*	Reinforcement	Ex-captive rehabilitated	5	17 Dec. 2020	(59)
9	*P. pygmaeus*	Wild-to-wild	Wild captured	10	During 2020	(60)
10	*P. pygmaeus*	Wild-to-wild	Recapture of wild OU	1	14 Jan. 2021	(61)
11	*P. abelii*	Wild-to-wild	Wild captured	1	30 Jan. 2021	(62)
12	*p. pygmaeus*	Wild-to-wild	Wild captured	1	30 Jan. 2021	(63)
13	*P. abelii*	Wild-to-wild	Wild captured	1	5 Feb. 2021	(64)
14	*P. abelii*	Wild-to-wild	Wild captured	1	9 Feb. 2021	(65)
15	*p. pygmaeus*	Wild-to-wild	Recapture of wild OU	1	10 Feb. 2021	(66)
16	*p. pygmaeus*	Reinforcement	Ex-captive rehabilitated	7	16 Feb. 2021	(67)
17	*p. pygmaeus*	Reinforcement	Ex-captive rehabilitated	3	16 Feb. 2021	(67)
18	*p. pygmaeus*	Wild-to-wild	Recapture of wild OU	1	22 Feb. 2021	(68)
19	*p. pygmaeus*	Wild-to-wild	Wild captured	1	10 Mar. 2021	(69)

*Translocations recorded between March 15, 2020 and March 14, 2021. Each translocation event is a release of one or more animals together at the same site on the same day. Event 9 covers 10 animals reported released in 2020 with no further detail provided on number of events or specific dates; however no public records of these translocations were found between January 1 and March 14, 2020, so these releases were assumed to have occurred March 15, 2020 or later. Orangutan is abbreviated as OU. BKSDA is Balai Konservasi Sumber Daya Alam, the government natural resources agency, a part of KSDAE. Recapture of wild OU are captures and rereleases of previously released wild orangutans. Three individuals are listed more than once as they were translocated multiple times (female “Maria” in events 2 and 3, male “Boncel” in events 4, 10 and 18, and female “Gisel” in events 12 and 15)*.

In March 2020, IUCN Primate and Wildlife Health specialist groups recommended halting all releases of great apes in light of the extraordinary risk posed by the potential transmission of COVID-19 from humans to non-human great apes (30, 33). The Indonesian government prohibited captive wildlife releases in mid-March 2020 to prevent disease transmission to wild populations (71, 72). On May 20, 2020, the Indonesian Ministry of Environment and Forestry released a circular lifting this moratorium and providing technical instructions for wildlife releases during the COVID-19 pandemic (73, 74). The circular stipulates that all animals, including wild individuals that have been near humans, must go through medical examination and receive a certificate of health from KSDAE prior to release, and that the number of personnel involved in the release should be limited to the smallest number feasible to safely conduct release activities (73). All wildlife rescues and releases are conducted by Indonesian government personnel from the KSDAE (75, 76). Results of the implementation of the circular were not publicly available.

**Figure 2:**
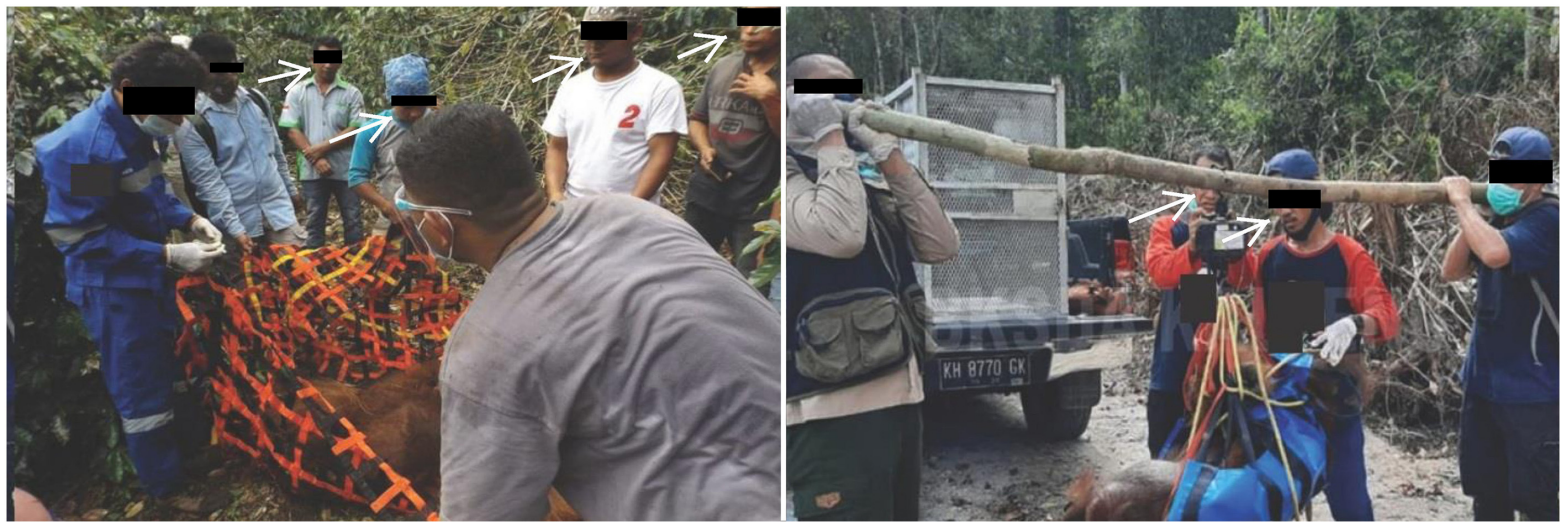
PPE use and human proximity in wild-to-wild orangutan translocations during COVID-19 pandemic. Pictures showing examples of mixed use of PPE and human proximity to orangutans during captures and releases of wild orangutans between March 15, 2020 and March 14, 2021. The orangutans are in the blue sling and the orange and black net, respectively. Identifiable human features and organizational logos are obscured to protect anonymity. Image credits from left to right: BBKSDA Sumatera Utara via KSDAE Top News blog http://ksdae.menlhk.go.id/assets/uploads/ou_sipirok2.JPG (70); BKSDA Kalimantan Tengah via Facebook https://www.facebook.com/BKSDAKalimantanTengah/posts/1365289040500502 (69).

**Figure 3:**
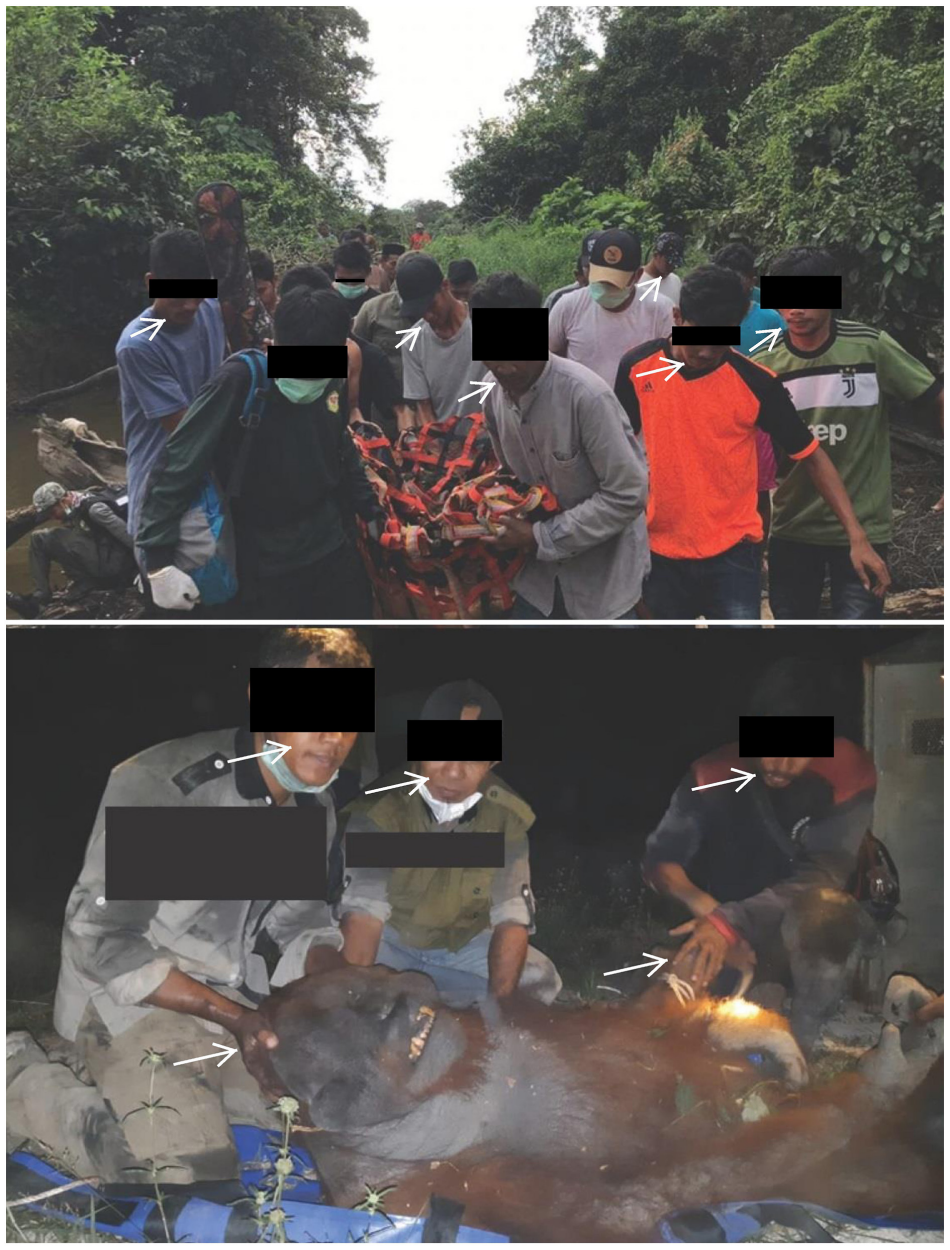
PPE use and human proximity in wild-to-wild orangutan translocations prior to COVID-19 pandemic. Pictures showing examples of mixed use of PPE and human proximity to orangutans during capture and releases of wild orangutans prior to March 15, 2020. The orangutan in the top photo is in the orange and black net. Identifiable human features and organizational logos are obscured to protect anonymity. Image credits from top to bottom: BKSDA Aceh via KSDAE Top News blog http://ksdae.menlhk.go.id/assets/news/Translokasi_ortu1.jpeg (78); BKSDA Kalimantan Tengah via KSDAE Top News blog http://ksdae.menlhk.go.id/assets/news/gambb.jpg (80).

While some safety measures have been taken to prevent the spread of COVID during translocations, these measures have been inconsistent and insufficient to avoid potential pathogen exposure risk to wild populations. Photographs in news and social media reports showed some precautionary measures such as personal protective equipment (PPE) were applied during the study period (57, 67, 77), but use was not consistent. Photographs show many people present at releases, often in direct contact with orangutans (Figure 2). Some examples include: (1) a wild-to-wild translocated *P. tapanuliensis* surrounded by seven people, four of whom were not wearing any PPE (70); (2) a wild-to-wild translocation during the captive wildlife release moratorium (March 23, 2020) with at least 10 people present, and three people wearing masks below their nose or mouth (52); and (3) two people next to an anesthetized wild-to-wild translocated orangutan, both with masks below their chins (69). Such situations also occurred in release records prior to COVID (78–80) (Figure 3). We could not assess the proportion of releases during the study period with adequate vs. inadequate protective measures because published photographs were not available for every translocation event, and when available do not capture every aspect of the entire procedure. However, the available images illustrate the reality of the disease transmission risks during orangutan translocation.

### Pathogen Risk Pathways in Orangutan Translocations

Pathogen transmission can occur via numerous pathways inherent in the orangutan rescue, rehabilitation and translocation process (Table 2). This includes consideration of transmission risks posed by exposure times, e.g., 15 min or longer for COVID-19 (39). The table addresses pathways for other pathogens of concern that can cause morbidity or mortality in orangutans, can be transmitted to wild orangutans, and have been identified in captive orangutans. Examples of these pathogens include members of the viral families Hepadnaviridae, Picornaviridae, Phabdoviridae (81, 82), bacteria such as *Streptococcus sp, Burkholderia pseudomallei* (82) and *Mycobacterium tuberculosis* complex (83), and parasite species including *Plasmodium sp*. (including strains that also infect other non-human primate species and humans) (84), *Entamoeba histolytica* and *Strongyloides sp*, that would all likely result from contact or proximity to humans (84). Pathways of zoonotic disease transmission between humans and orangutans or among orangutans include direct and indirect contacts (abiotic transfer via fomites and other transmission ways), and animal vectors.

### Problem Description

Based on evidence that the problem can potentially occur through a variety of pathways inherent in the orangutan translocation process, we developed a problem description (Table 2). We included data presented in this study, along with contextual information on conservation, socioeconomic, political, and ethical considerations from published literature (see discussion) to answer the problem description questions posed by OIE and IUCN (36). This problem description highlights a large number of data gaps and assumptions, indicating that a qualitative method of assessment based on the precautionary principle will likely be needed in the first iteration of a DRA for orangutan translocation.

### Qualitative Logic Matrix for SARS-CoV-2 Risk in Orangutan Translocation

**Figure 4:**
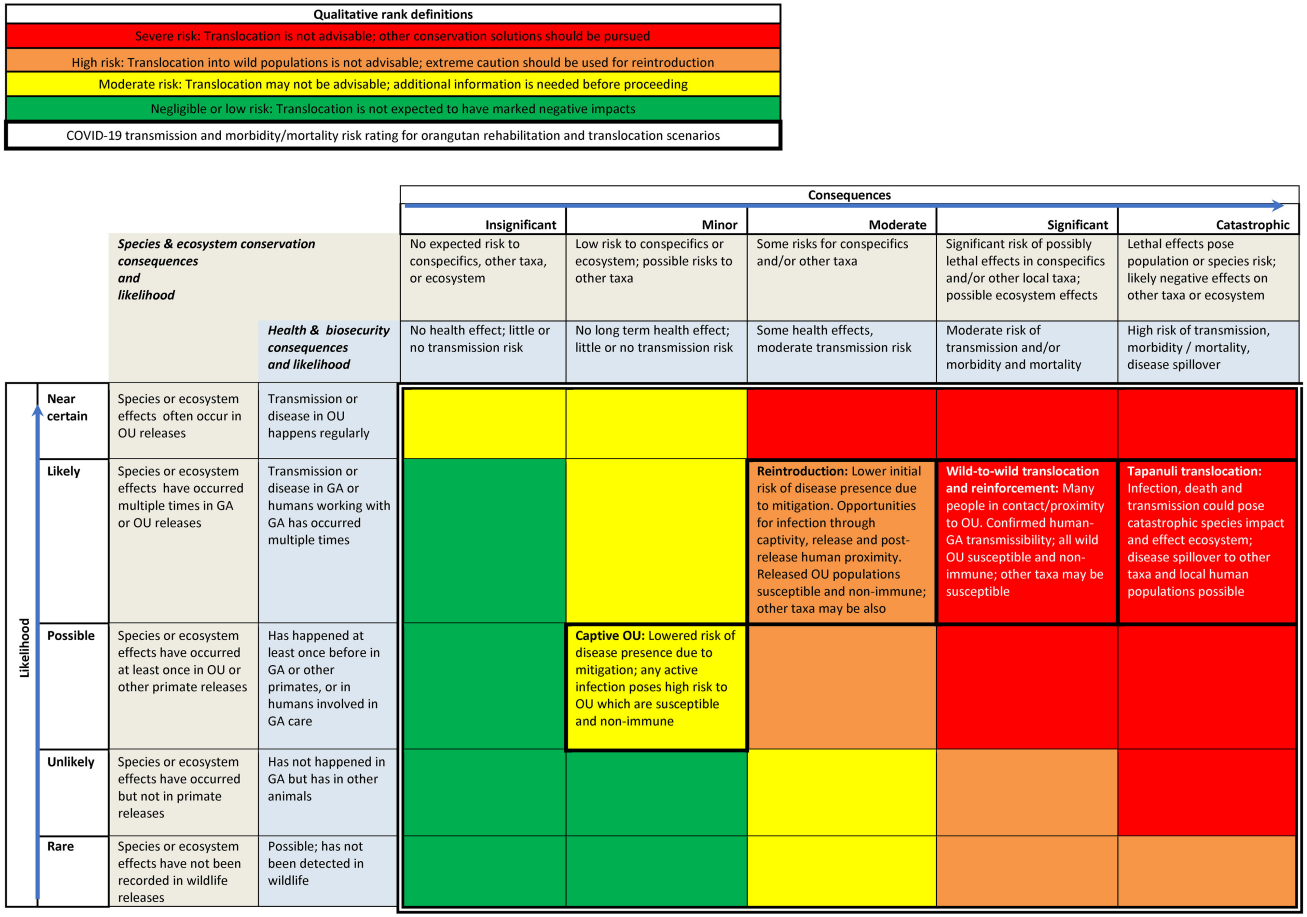
Qualitative risk matrix for orangutan translocation using COVID-19 example. OU = orangutans; GA = great apes. Colored cells inside double lines denote overall qualitative rank. Consequence definitions are presented in the two cells beneath each category name, with species and ecosystems in the top cell and health and biosecurity in the cell below. Likelihood category definitions are presented in the two cells to the right of each category name, for species and ecosystems and then for health and biosecurity from left to right. Cells relevant to OU translocation scenarios have a thick border and list factors considered in risk ranking.

We used the data summarized in the problem statement to make a qualitative risk assessment matrix that can be used by practitioners to consider overall threats associated with orangutan translocations (Figure 4). Likelihood for transmission from humans multiplies as more people interact with or are in proximity to the orangutan, and as exposure times lengthen. We also considered available information on mitigation measures and uncertainties, all of which modify likelihoods and consequences.

The risk of catastrophic consequences is highest for wild-to-wild translocation of Tapanuli orangutans. Tapanuli orangutans are the world's rarest great ape species, with a population of <800 individuals under significant threat from human activities (8). The capture and release of these orangutans puts them at risk of exposure to the virus from infected humans. COVID-19 is uncontrolled in Indonesia at the time of writing, with only 17% of the population vaccinated (85), and it is possible that humans in proximity to translocated animals will not use adequate disease risk mitigation measures (Figures 2, 3). Translocation of these animals into a small, geographically constrained wild population that is susceptible and non-immune to COVID-19, and already at serious risk of extinction, poses marked risks. Slightly lower but still severe risk is posed by wild-to-wild translocation of *P. abelii* and *P. pygmeaus*, or their release to reinforce wild populations, again due to known susceptibility of the species to the COVID-19 pathogen, likely exposure of translocated orangutans to humans without protective measures, and that wild and formerly released animals are non-immune.

Reintroduction of rehabilitated ex-captive orangutans is somewhat lower risk due to extensive protective measures available for captive animals and staff. Nonetheless, the risk remains high due to pathways for infection during captivity or release, or following release (at provisioning or observation sites), and subsequent risk of pathogen transmission to susceptible and non-immune conspecifics at the release site. Captive orangutans pose a moderate risk overall, as protective measures can be employed consistently. As with reintroduction, opportunities for infection during captivity still persist due to human contact and proximity.

## Discussion

Problem descriptions such as the one presented here can be useful tools to help forge agreement among stakeholders, which is vital for successful risk communication and advancing the DRA process (36, 38). Anecdotally, the lack of a problem statement is where a lot of DRA processes fail: when stakeholders cannot agree on the problem, it is more challenging to convince decision makers to follow the DRA recommendations for risk management (Unwin, unpublished data). This paper poses questions that must be answered by the orangutan translocation community before progress can be made toward a full DRA. The context and drivers of orangutan translocations further complicate potential disease and conservation risks by creating pressure for translocation as an ostensibly rapid, short-term solution to protect orangutans.

Despite the popularity of translocation, survival rates of released orangutans are not well known. Orangutans are difficult to follow and radio collars have not worked well with orangutan anatomy, so Very High Frequency (VHF) implants are used for tracking rehabilitated and released animals (86). Wild-to-wild translocated orangutans are generally microchipped for identification but almost never monitored post release (4). This means their adaptation and survival following release are unknown unless they return to the areas where they were originally captured (see Table 3). Estimated survival rates of rehabilitated and released orangutans range from 6 to 80%, but 40–95% of these released orangutans are not re-encountered after release (4). Researchers also note that as many as 1,200 orangutans released in Kalimantan, Indonesia may have disappeared or died following release, but poor record keeping and reporting makes exact numbers impossible to verify (87).

**Figure 5:**
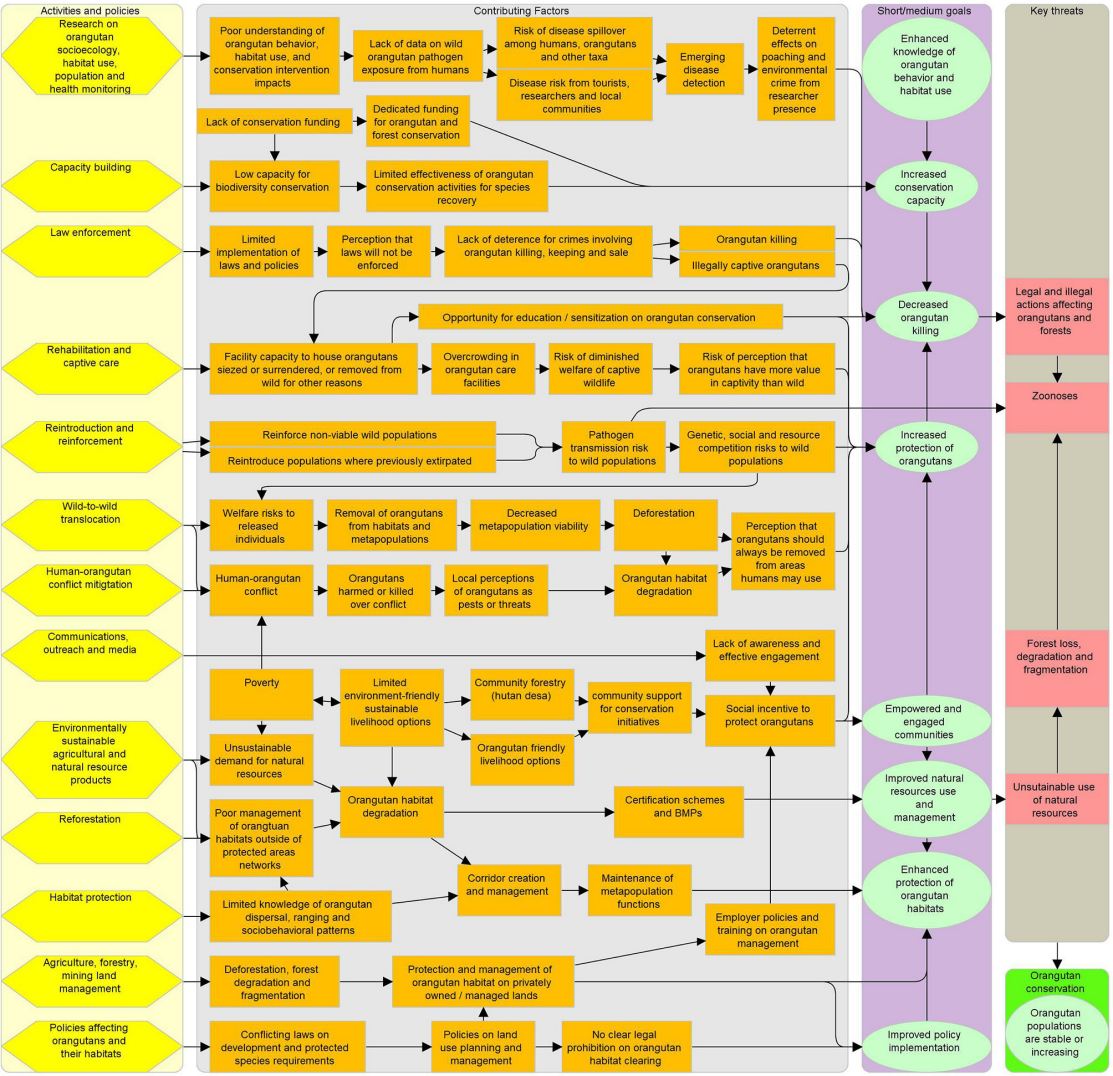
Orangutan conservation complexity. Cells in far left column are activities and policies affecting orangutans. Central section cells describe positive and negative factors that affect orangutan conservation outcomes. The second column from the right shows the interim goals of activities and policies. Key threats that activities and policies aim to address are in the far right column. Arrows indicate relationships between cells.

The 2007–2017 Indonesian national Conservation Strategies and Action Plan for Orangutans mandated release of all orangutans from rescue centers by 2015, which proved impossible given intake and birth rate. A draft 2019–2029 plan for Indonesian orangutan conservation called for all releasable orangutans to be translocated by 2024 (4). Though similarly unachievable due to lack of suitable release sites for the more than 1,000 orangutans in captive care, these plans create significant political pressure to release as many animals as possible. Some practitioners state that overcrowding at centers or pressure from donors is a rationale to continue releasing rehabilitated ex-captive orangutans (72, 74, 88). Holding orangutans in captivity is costly, and most centers are already full or over-capacity (72, 89, 90). The 2019 plan describes wild-to-wild translocation as a last resort option, but in practice both NGO and government officials publicly request that local residents report any orangutans they see in or near areas of human use, and practitioners note that community members view it as the government's job to move any such orangutans (4) (Jaya, unpublished data). A diagrammatic view of the complexity of orangutan management and conservation is presented in Figure 5.

### Context and Drivers of Orangutan Translocation

Populations of all three species of orangutans are declining, primarily due to forest loss and killing (6). The underlying drivers of these threats complicate efforts to conserve orangutans. Poverty and well-being are significant concerns across much of Indonesia, particularly in rural areas with limited access to markets (91). Expansion of palm oil production, including in orangutan range, has been championed by industry and government as a solution for poverty alleviation, despite mixed results for human livelihoods and well-being (92). Results for orangutans, on the other hand, have been markedly poor (40, 41). However, conservation and management of orangutan populations within forest reserves and forested patches in palm oil plantations is possible if killing is avoided (5, 43, 93).

Where forests are cleared for agriculture, agro-forestry, natural resource extraction, or human settlements, orangutans are often driven out of their original habitats or have easier access to human crops (94–96). This can lead to conflicts with people when orangutans are found crop foraging or using human-occupied areas. Orangutans are often injured or killed by humans in these situations (4, 97). Orangutans are also killed for food, for traditional medicine, or to obtain live infants for pets (98).

Capturing infant orangutans nearly always necessitates killing the mother (99). Although illegal, keeping infant orangutans as pets is common in Indonesia, as is injury or harassment of wild orangutans found in human-occupied areas (4, 99). On average, more than 115 wild or illegally captive orangutans are being surrendered, confiscated or otherwise rescued by wildlife rescue organizations and government annually (4). Many Indonesians who buy or take an infant orangutan are motivated to “save” or care for the animal, although most privately held animals are rescued from malnourishment and often horrific conditions (4, 100).

Against this background of poverty, strong demand for forest lands, high killing rates and consistent local demand for pet orangutans, translocation is often marketed to the public as a means to “save” orangutans (4). Investments into orangutan rescue, rehabilitation and release total millions of dollars annually, but have not halted the continued decline in species populations (44), although these investments have prevented losses from being even greater (Santika et al., *in prep*). Orangutan releases are considered a conservation necessity by some (101), while others note that releases do not address the taxon's most urgent conservation needs, namely preventing deforestation and fragmentation of natural forest habitats, and forestalling the loss of wild orangutans by addressing killing and capture (9, 44). Rehabilitation and release also cost more than 12 times as much per orangutan as improved habitat protection (45). Nonetheless, releasing captive orangutans back to the wild is considered the only ethical option by many practitioners (46). Orangutan release also presents an ostensibly “quick and easy solution,” while habitat protection or community engagement to address killing requires longer term investment and effort, particularly in lowlands where stakeholders with complex and vested interests exist (Santika et al., *in prep*). Reintroduction of orangutans into historical range but outside current distribution has resulted in establishment of two new Sumatran orangutan populations, which are projected to become self-sustaining over time if releases continue over the next ten years and habitat loss and killing are prevented (102). Notably, habitat incursion and human-orangutan conflict are pressing concerns in at least one of these areas (103). Rehabilitated ex-captive and wild-to-wild translocated orangutans have also been released to reinforce viable and some small non-viable wild populations in both Kalimantan and Sumatra (4).

Orangutans metapopulations are multiple interacting individual orangutan populations bounded by geographic barriers. These metapopulations are projected to continue declining despite release activities, due to habitat fragmentation, forest loss and orangutan killing or removal (102). This is partly explained by the fact that releases do not represent net additions of orangutans to wild populations. Most ex-captive orangutans represent a net loss of at least two wild orangutans from the source population (the mother, likely killed, and the captured infant), with the rehabilitated former infant released after years of rehabilitation into another population (4). Wild-to-wild translocations thus represent a lateral movement comprised of removal of the wild orangutan from one local population, and its release into another.

Orangutan behavior and socioecology pose challenges for rehabilitation and survival of released orangutans, further complicating the potential for releases to boost wild populations (87). Wild adults of both sexes face difficulties in establishing a home range and adapting socially if translocated to a new location where the local orangutan population is unfamiliar to them. Wild-to-wild translocations are a particular concern in this regard, as they capture and remove wild orangutans from their metapopulation, disrupting the breeding and social structures of dispersed males and resident females (5). Importantly, different forest types within orangutan range differ in their seasonal, annual and spatial distribution of fruit and other food resources (104, 105), meaning orangutans may not know where and when to find important foods if they are translocated to unfamiliar locations (4). This may explain why wild-to-wild translocated orangutans often return to their capture site from following release, resulting in some animals being recaptured and moved multiple times (Table 3). Finding food in an unfamiliar habitat could also pose energetic stresses. Orangutans can exhibit decreased body weights and depleted fat stores during periods of low fruit availability (106). Wild orangutans with low body weights found foraging in oil palm plantations or other human crops are often considered to be starving and in need of being moved to new habitats, but instead may in some cases be going through a natural cycle of muscle catabolism during low fruit availability.

### Disease Risks

All this wicked complexity (9, 11) is happening in a context of limited information on orangutan disease surveillance and risk management. Orangutan veterinarians and rescue, rehabilitation and release NGOs and government agency staff are part of a long-running capacity development project, the Orangutan Veterinary Advisory Group (OVAG). OVAG provides capacity building on disease diagnosis and treatment, surveillance, risk management, and development of DRAs. Studies have been conducted on outbreaks in orangutan rehabilitation facilities (107, 108), and there have been instances of orangutans released with transmissible pathogens (109). Surveillance for wild orangutan diseases, zoonotic pathogen transfer, disease spillover and emerging diseases is limited, and indeed most wild populations are not surveilled. We are not aware of any DRA for orangutan translocation, and there is a paucity of data available on the management and risk abatement of pathogens that could infect orangutans.

Practitioners may perceive zoonotic disease transmission as less likely for orangutans than for other great apes, because of orangutan's semi-solitary lifestyle, more arboreal behavior in natural conditions, and the lack of clear examples of diseases wiping out orangutan populations following releases (23, 24, 110). But orangutans do come to the ground (111) and can contract human carried diseases, especially those transmitted via the fecal-oral or airborne transmission routes, respiratory viruses and bacteria in particular (110). Respiratory ailments sinusitis, airsacculitis and pneumonia are of particular concern for orangutans (112, 113). Further, males may travel among wild populations to find females (5, 105). This underscores the suggestion that individual orangutans can be disease superspreaders among conspecifics (114). Importantly, orangutans captured due to their proximity to humans may have already been exposed to human diseases. For example, orangutans have been observed using small puddles and human-constructed drains as water sources, which are highly likely to be contaminated with human waste (Oram, unpublished data) and which can also facilitate infections such as malaria from insect vectors (108).

Active COVID-19 infections are circulating among humans in Indonesia at a reported rate of 2,931 average daily cases and 182 average deaths daily as of September 2021 (85). At least one COVID-19 variant of concern, the more transmissible Alpha (formerly B.1.1.7), was verified in Indonesia as of March 16, 2021 (115). Although orangutan-specific consequences of SARS-CoV-2 remain unknown, extensive contact with caretakers during rehabilitation, the number of people involved in orangutan care and release, and occasional contact or proximity with humans through post-release monitoring, field research activities, provisioning or tourism potentially exposes orangutans to becoming asymptomatic carriers of contagious diseases, including SARS-CoV-2 (21–24, 31). Humans can transmit COVID-19 several days prior to being symptomatic, as occurred with captive gorillas (116). Transmission from humans to apes is thus classified as “likely” (Figure 4). Although rescue practitioners regularly use disease mitigation measures, wild-to-wild translocated orangutans are exposed to extensive human interactions that likely include humans without PPE or testing. Rehabilitation of ex-captive orangutans includes episodes of frequent exposure to human interaction over long periods (4), but likely with consistently applied mitigation measures, hence with lower overall risk. Nevertheless, there are unknowns for all releases including the potential for people to transmit disease even while using PPE or after vaccination.

Rescue centers mitigate many risks via disease surveillance and testing for likely zoonotic diseases in rehabilitated ex-captive orangutans, and government policies require proof of disease testing results, including for SARS-CoV-2, prior to release of ex-captives. Specifically regarding COVID-19, orangutan rescue organizations reported making significant efforts and financial outlays to implement precautionary protocols such as regular COVID-19 antigen testing for staff, use of PPE, and closures to tourism and volunteers (74, 117–125). Rescue organization personnel are also getting vaccinated against COVID-19. Vaccination has been successful in preventing COVID-19 infections in captive populations of black-footed ferrets, an endangered species susceptible to the disease (126). In the United States some captive populations of orangutans and other great apes have been vaccinated (116) and vaccination of captive Indonesian orangutans may become an option. However, species-specific healthcare and disease risk management expertise varies across orangutan facilities and personnel, as do efficacy of diagnostic tests and vaccines, and exposure of orangutans to humans. In particular, wild orangutans captured and translocated during the COVID-19 pandemic may be at greater risk of exposure per the World Health Organization's (WHO) definition (39): During the process of capture, examination, transport, and release, these animals are in direct contact with humans and at distances of <2 m from people, many without any PPE (Figures 2, 3), for more than 15 min. In some cases, there are large numbers of people without PPE in contact or close proximity to the animals (Figures 2, 3).

It is likely that any infected orangutans released into wild or reintroduced populations could spread SARS-CoV2 among conspecifics, thus consequences qualify as at least “significant” (Figure 4). SARS CoV2 affects multiple species, meaning likelihood of further species spillover is high (31, 127). Such spillover could pose catastrophic consequences to species, ecosystems and potentially humans (Figure 4). These considerations are applicable to any other transmissible diseases that could affect orangutans.

## Conclusion and Recommendations

Our study suggests that orangutan translocations can pose considerable infectious disease transmission and species conservation risks. Pathways exist for released orangutans to potentially transmit COVID-19 to susceptible and non-immune wild populations. Captive situations with effective biosafety protocols including staff testing are expected to have low prevalence of transmissible diseases among associated humans and thus overall lower risk. Wild-to-wild translocations have increased disease exposure during human-orangutan conflict situations and during rescue due to increased incidence and duration of contacts with humans.

The IUCN Primate Specialist Group and great ape disease risk experts discourage translocating great apes during the COVID-19 pandemic, and particularly advise against wild-to-wild translocation as it could pose risks to wild conspecifics (30, 128). Review of available data indicates that practitioners generally made significant efforts to manage disease risks while continuing to undertake wild-to-wild translocations, reintroductions, and reinforcements at reduced levels during the COVID-19 pandemic. However, we identified circumstances of extended human-orangutan contact and proximity that create additional disease transmission pathways.

Most orangutans in Kalimantan, Indonesia, live outside protected areas, and orangutans are frequently killed, injured or displaced by humans and human activity (44, 97). Practitioner's concern for the welfare of the individual orangutans and pressure from local communities to avoid further interactions or conflict with the orangutans drive wild-to-wild translocation decision making (46). Wild-to-wild translocations account for 64% of orangutans released between March 15, 2020 and March 14, 2021 during the COVID-19 pandemic (Figure 1). Researchers found most wild-to-wild translocated orangutans were healthy based on physical examination when captured, suggesting they may have survived in their original habitat, even in small forest patches, if protected from killing (4, 5). Other individuals were rescued from situations of imminent harm like forest fires, human-inflicted injuries, and forest clearing (129–132). Wild-to-wild translocations are conducted without disease testing to facilitate quick release of animals (15, 78–80) (Jaya unpublished data). This lack of testing highlights the disease risks posed to Critically Endangered orangutan populations by frequent wild-to-wild translocations, especially during a pandemic. These risks outweigh the potential species conservation benefits of moving wild individuals. This is particularly the case for Tapanuli orangutans. One Tapanuli orangutan was translocated into a wild population during the pandemic, despite an estimated total species population of 760 animals or fewer, and the concomitant extreme risk posed by potential disease transmission into such a limited population. This animal was previously translocated in 2019 and had moved back into an area around humans (42).

We recommend that the next step for orangutan translocation stakeholders is to convene to discuss and accept the problem statement presented here. Subsequently a formal DRA process should be conducted for orangutan translocation including the risk pathways identified in this study. The goal will be to achieve a consensus among stakeholders on the relative disease risks as well as agreement on what level and degree of risk that they and their respective organizations are willing to accept. Due to high risks and high uncertainty of the pathogen transfer potential and outcomes of orangutan translocations identified in this problem description, the precautionary principle is indicated. Application of this principle means assuming the worst case of high mortality in captive, reintroduced, and wild orangutan populations unless or until evidence indicates otherwise. It requires preventing avoidable risks and focusing on biosecurity to break the transmission cycle. Releases are an unsuitable option in situations where there risk of catastrophic species and ecosystem effects and disease spillovers is greater than negligible or low (Figure 4). While zero risk is impossible, circumstances with manageable and acceptable risks pose reduced threats to species survival, ecosystem function, and disease spillovers.

Where reintroduction, reinforcement or wild-to-wild translocations are determined to be the best conservation option, we assert that minimum disease mitigation measure requirements should be enforced. These should stipulate use of effective PPE that fit tightly and cover the mouth and nose (33), worn by all persons, even if vaccinated. Vaccinated persons can still carry and multiply the virus, and vaccines may be less effective in preventing disease transmission of the delta variant (133) and potential future variants. When cost or scarcity may make it difficult for release practitioners and community members to obtain adequate PPE, this should serve as a reason to forego translocations rather than to continue with inadequate mitigation measures. The number of people present at releases should be strictly limited. Samples (feces, urine, blood, and orifice swabs) should be taken from all translocated orangutans (134), regardless of apparent health status, and all should be tested for diseases of concern, including COVID-19. Diagnostic tests for humans and orangutans should be selected for proven detection effectiveness. The risks to wild captured orangutans rise with increased human contact, thus captured animals suitable for release should be translocated as quickly as possible to minimize exposure and limit stress and behavior changes.

A One Health approach to disease risk mitigation, public health engagement and conservation in line with the One Health approach is critical, including: (1) health surveillance of wild, captive and released orangutans, and information sharing among all stakeholders; (2) collaboration among government, local communities and NGOs to address health and biodiversity conservation; (3) a DRA conducted with orangutan conservation and translocation stakeholders and wildlife health experts; and (4) investment in education and policy that recognizes the direct dependence of human health on functional ecosystems and biodiversity (36, 135). Improved law enforcement to address orangutan killing, trade, and clearance of orangutan habitats in agricultural concessions, addressing negative human interactions with orangutans, and protection of large intact forests and forest fragments have been identified as orangutan conservation priorities (4, 5, 44).

As humans expand their presence across orangutan range, close proximity and contact between humans and orangutans will continue to increase. While translocations are one possible tool for addressing extinction risk, renewed focus on preventive action to protect habitats and mitigate negative human-orangutan interactions is needed.

Decisions about interventions for individual orangutans in undesirable situations involve complex ethical considerations, but we encourage adherence to the IUCN guidelines to weigh net risks and benefits to species conservation (1). In extraordinary circumstances, wild-to-wild translocation will be the only option, such as when orangutans cannot escape forest fires, or people are likely to kill them and cannot be swayed by legal consequences or alternative solutions. Otherwise, outreach measures to protect orangutans in place rather than moving them should be pursued. Numerous resources exist for addressing human-wildlife interactions (136), some specific to great apes or to orangutans (137–139). Solutions that provide financial benefits, employment opportunities, or improved livelihoods should be determined in collaboration with local communities to address their specific needs. While there are few counterfactual-based studies on orangutan conservation interventions, demonstrated effective strategies include health care services tied to illegal logging reductions (140) and community forest management initiatives (141). In areas where orangutans need to move between forest blocks within agricultural landscapes, crops unpalatable to orangutans, like shade-grown coffee, may be helpful (142). Other solutions to forestall potentially negative interactions could include engaging local community members to serve as “orangutan guardians,” indirect incentives such as infrastructure and civic facilities (103), or direct financial incentives (143). Rescue centers, research centers, and locally-based NGOs are crucial to developing solutions, as they have longstanding relationships with surrounding communities, and often serve as significant source of local employment (141).

## Data Availability Statement

The original contributions presented in the study are included in the article/supplementary material, further inquiries can be directed to the corresponding authors.

## Author Contributions

JS, MA, EM, and SW conceived the idea for the paper. SU, DT, MA, and JS developed the risk assessment. JS collected the data, developed the figures, and wrote the manuscript. RJ, FO, TS, and MA contributed data and observations. EM and MV helped with figure design. All authors contributed to revising the manuscript text and figures, read, and approved the final version of the manuscript.

## Funding

We thank the United States Fish and Wildlife Service Great Ape Conservation Fund for financial support (Grant No. F17AP01081). The funders had no involvement in study design, in the collection, analysis and interpretation of data, or in the writing of the paper and the decision to submit the article for publication.

## Conflict of Interest

The authors declare that the research was conducted in the absence of any commercial or financial relationships that could be construed as a potential conflict of interest.

## Publisher's Note

All claims expressed in this article are solely those of the authors and do not necessarily represent those of their affiliated organizations, or those of the publisher, the editors and the reviewers. Any product that may be evaluated in this article, or claim that may be made by its manufacturer, is not guaranteed or endorsed by the publisher.
